# Multiple Sclerosis and *Clostridium perfringens* Epsilon Toxin: Is There a Relationship?

**DOI:** 10.3390/biomedicines12071392

**Published:** 2024-06-23

**Authors:** André Huss, Franziska Bachhuber, Cécile Feraudet-Tarisse, Andreas Hiergeist, Hayrettin Tumani

**Affiliations:** 1 Department of Neurology, University Hospital Ulm, 89081 Ulm, Germany; andre.huss@uni-ulm.de (A.H.); franziska.bachhuber@uni-ulm.de (F.B.); 2 CEA, INRAE, Medicines and Healthcare Technologies Department (DMTS), SPI, Paris-Saclay University, 91191 Gif-sur-Yvette, France; 3 Institute of Clinical Microbiology and Hygiene, University Medical Center, 93053 Regensburg, Germany

**Keywords:** multiple sclerosis, *Clostridium perfringens*, *C. perfringens* epsilon toxin, microbiota

## Abstract

Recent research has suggested a link between multiple sclerosis and the gut microbiota. This prospective pilot study aimed to investigate the composition of the gut microbiota in MS patients, the presence of *Clostridium perfringens* epsilon toxin in the serum of MS patients, and the influence of disease-modifying drugs (DMDs) on epsilon toxin levels and on the microbiota. Epsilon toxin levels in blood were investigated by two methods, a qualitative ELISA and a highly sensitive quantitative ELISA. Neither epsilon toxin nor antibodies against it were detected in the analyzed serum samples. 16S ribosomal RNA sequencing was applied to obtain insights into the composition of the gut microbiota of MS patients. No significant differences in the quantity, diversity, and the relative abundance of fecal microbiota were observed in the gut microbiota of MS patients receiving various DMDs, including teriflunomide, natalizumab, ocrelizumab, and fingolimod, or no therapy. The present study did not provide evidence supporting the hypothesis of a causal relationship between *Clostridium perfringens* epsilon toxin and multiple sclerosis.

## 1. Introduction

Multiple sclerosis (MS) is a chronic inflammatory disease of the central nervous system (CNS) that primarily affects young adults and often leads to accumulating neurological symptoms and disability. Cellular components of the immune system, including B and T lymphocytes, play a pivotal role in the pathogenesis of MS and are the target of current disease-modifying therapies (DMTs). Although the cause of MS is still largely unknown, it has been established that genetic, environmental, and lifestyle risk factors contribute to the complex pathophysiological cascade in MS.

Recent research has suggested a link between MS and the microbiome, particularly the gut microbiota. The microbiota are a collection of microorganisms that live on and inside the human body, including their genome and activity [1]. The gut microbiota encompass the collection of microorganisms that live in the digestive tract. These microorganisms play a critical role in many physiological processes, including digestion, immune function, and metabolism, as well as CNS development and function [2]. As the gut microbiota play an important role in regulating immune function, a disbalance in the gut microbiota may contribute to the pathogenesis of MS.

When comparing the gut microbiota of MS patients and healthy subjects, Thirion and colleagues found substantial microbiome alterations in MS patients that were linked to blood biomarkers of inflammation [3]. In a mouse model of relapsing–remitting, spontaneously developing experimental autoimmune encephalomyelitis (EAE), the presence of non-pathogenic commensal microbiota was a prerequisite for the induction of EAE in mice [4]. Furthermore, in transplantation experiments using microbiota obtained from monozygotic twin pairs discordant for MS, the microbiota from MS-affected twins exhibited a significantly higher propensity for inducing autoimmunity in spontaneous autoimmune encephalitic mice as compared to microbiota derived from their non-MS twin siblings [5]. 

One specific bacterium that has come into focus in connection with MS is *Clostridium perfringens*. *C. perfringens* is a Gram-positive, spore-forming anaerobic bacterium that is commonly found in the soil and in the intestines of animals, including humans [6,7]. It is known to cause a variety of diseases, including gas gangrene, wound infections, food poisoning, and enterotoxaemia. More recently, it has been suggested that *C. perfringens* may play a role in the development of MS. One of the toxins produced by *C. perfringens* (*C. perfringens* type B and D) is epsilon toxin. This protein toxin is a potent pore-forming neurotoxin that is able to cross the blood–brain barrier [6]. Among other modes of action, in the CNS, epsilon toxin causes blood–brain barrier disruption and has detrimental effects on neurons, oligodendrocytes, and astrocytes [7]. In laboratory studies, epsilon toxin has been shown to cause demyelination, a process that is also observed in MS [8,9].

In 2013, the isolation of *C. perfringens* type B from an MS patient was reported [10]. This finding, together with the known CNS-tropism of *C. perfringens* epsilon toxin, prompted the authors to investigate a potential connection between epsilon toxin and multiple sclerosis. A study in a US population found a higher frequency of immunoreactivity against epsilon toxin in the cerebrospinal fluid and serum when comparing MS patients to healthy controls [10]. Another investigation in a UK population of relapsing–remitting MS, clinically isolated syndrome (CIS), and optic neuritis (ON) patients reported a higher frequency of antibodies against *C. perfringens* epsilon toxin in serum as compared to controls [11]. 

Teriflunomide is a disease-modifying therapy approved for the treatment of relapsing forms of multiple sclerosis (RRMS). Its mechanism of action involves inhibiting the enzyme dihydroorotate dehydrogenase, which is involved in the synthesis of DNA in rapidly dividing cells, including lymphocytes and mononuclear cells [12]. Teriflunomide has been shown to alter the composition of the gut microbiome in animal studies and may also have an impact on the microbiome [13]. It was hypothesized that teriflunomide might have a direct influence on the bacterial counterpart (O-GlcNAcase) of human dihydroorotate dehydrogenase and acts on the *C. perfringens* population in the intestine. 

The aim of this study was to further investigate the gut microbiota in MS patients. Due to the presumed effect of teriflunomide treatment on the gut microbiota, especially on *C. perfringens*, we were further interested in a potential influence of DMTs with teriflunomide on serum epsilon toxin levels.

## 2. Materials and Methods

### 2.1. Ethical Approval

This study was conducted in accordance with the tenets of the Declaration of Helsinki and approved by the Ethics Committee of Ulm University (approval number 412/17). All patients provided written informed consent.

### 2.2. Patients

Forty-one MS patients (27 female, 14 male) with a median age of 42 years (range 19–60 years) were enrolled. Patients visiting for diagnostic or treatment purposes were recruited from the University Hospital Ulm, department of Neurology, and the Specialty Clinic Dietenbronn. Exclusion criteria were CRP values > 1.0 mg/dL, treatment with corticosteroids within the last four weeks, or treatment with mitoxantrone or azathioprine within the last 12 months.

### 2.3. Sample Collection

Stool samples were self-collected by patients in their home environment using the OMNIgene GUT system, which provides sample stability for up to 60 days at room temperature (Steinbrenner Laborsysteme GmbH, Wiesenbach, Germany, Cat. Nr. OM-200). After receipt, samples were stored at −80 °C until analysis. A total of 37 stool samples were available from 36 patients (due to lack of stool sample returned, incorrect specimen collection, or withdrawn consent in the remaining patients). For this project, no additional collection of blood was performed. Serum samples, collected for diagnostic purposes, were included in the analysis. A total of 38 serum samples were available from 34 patients. 

### 2.4. Microbiome Analysis

Stool samples were shipped to microBIOMix GmbH (Regensburg, Germany) for 16S rDNA-based microbiome analysis [14]. The analysis was performed according to the company’s standard procedures. Briefly, DNA was extracted from stool samples, and the V1V3 region of the 16S rRNA gene was amplified. Amplicons were sequenced using the Ion Torrent GeneStudio S5 Plus platform (Thermo Fisher Scientific, Waltham, MA, USA) and the resulting reads were processed as previously described [15,16]. In addition, the total bacterial load was quantified from stool-extracted DNA by real-time PCR quantification of bacterial 16S rRNA copy numbers [17]. Microbiome sequencing data were analyzed in R using the dada2 pipeline. The pairwise adonis test was used to assess statistical differences of beta diversity between treatment groups. Differential abundance analysis was applied to identify statistically different taxonomic features between groups by calculating linear discriminant analysis effect sizes (LefSe) using the lefser package V1.14 [18].

### 2.5. Epsilon Toxin Analysis: Quantification of and Immunoreactivity against Epsilon Toxin

Serum samples were analyzed for the presence of epsilon toxin using a qualitative sandwich ELISA (Bio-X Diagnostics, Rochefort, Belgium) according to the manufacturer’s instructions. Additional positive control samples were included using isolated epsilon toxin from *C. perfringens* (obtained from Institute Pasteur, Paris, France) with a final concentration of 25 and 2.5 ng/mL. Serum samples were furthermore analyzed by Service de Pharmacologie et d’Immunoanalyse (SPI, Gif Sur Yvette Cedex, France), where a highly sensitive immunoassay for the detection of clostridial epsilon toxin in body fluids was recently developed [19]. The limit of detection was defined as the lowest concentration giving an absorbance signal greater than the mean plus 3 standard deviations from negative commercial human control serum replicates (test replicate #1: one commercial human serum (octuplicate); test replicate #2: four different commercial human sera used (tetraplicate for each serum); test replicate #3: triplicate for 5 different commercial human sera). The limit of quantification was defined as the lowest concentration giving an absorbance signal greater than the mean plus 10 standard deviations from negative commercial human control sera replicates (same test). Positive control samples were included with standard curve of isolated epsilon toxin from *C. perfringens* type D strain NCTC2062 (Institute Pasteur, Paris, France) spiked in negative commercial human control sera. All sera were diluted 3-fold in EIA buffer (100 mM potassium phosphate buffer pH 7.4, 0.1% bovine serum albumin, 0.15 M NaCl, and 0.01% sodium azide) containing a final 10% heterophilic blocking reagent. The sandwich ELISA was carried out as previously described [19] with slight modifications: detection was performed with biotinylated PεTX6 (100 µg/mL in EIA buffer for 2 h) followed by 30 min incubation with poly-horseradish peroxidase-labeled streptavidin. After the addition of a tetramethylbenzidine-containing substrate solution for 30 min, 2 M sulfuric acid stopped the reaction and absorbances were read at 450 nm. Furthermore, immunoreactivity towards epsilon toxin was analyzed at SPI by ELISA. Briefly, plates were coated with 5 µg/mL epsilon toxin (Institute Pasteur) and incubated overnight at 4 °C with 100-fold diluted patient sera in 100 mM TrisHCl buffer pH 8.0, 5% milk, 150 mM NaCl, 0.5% tween20, 0.25% CHAPS, and 0.01% sodium azide (Tris-milk buffer). Immunoreactivity towards epsilon toxin was then detected with biotinylated anti-human IgA, IgM, or IgG (IgG1, IgG3, IgG4 or IgG1, IgG2, IgG3, IgG4), monoclonal antibodies, and acetylcholinesterase-labeled streptavidin revealed by Ellman’s colorimetric method at 414 nm after 1 h [20]. Limits of detection and quantification were calculated as described above (test replicate #1: one commercial human serum used for negative control (octuplicate); test replicate #2: tetraplicate of five different commercial human control sera).

Ig/epsilon toxin immunocomplexes were investigated at SPI by a sandwich ELISA. Briefly, plates were coated with a mix of 3 murine anti-epsilon toxin monoclonal antibodies (PεTX5, PεTX6 and PεTX7 [19]), incubated for 1h with 30-fold-diluted patient sera in Tris–milk buffer, and detected using peroxidase-coupled goat anti-human antibodies revealed with ABTS diammonium salt at 414 nm after 30 min. Five different commercial human sera were applied as negative control. Each commercial serum was analyzed in quadruplicate. A serum absorbance signal greater than the mean absorbance plus three standard deviations from the negative commercial human control sera was set as the limit of detection.

## 3. Results

### 3.1. Microbiome Analysis

**Figure 1:**
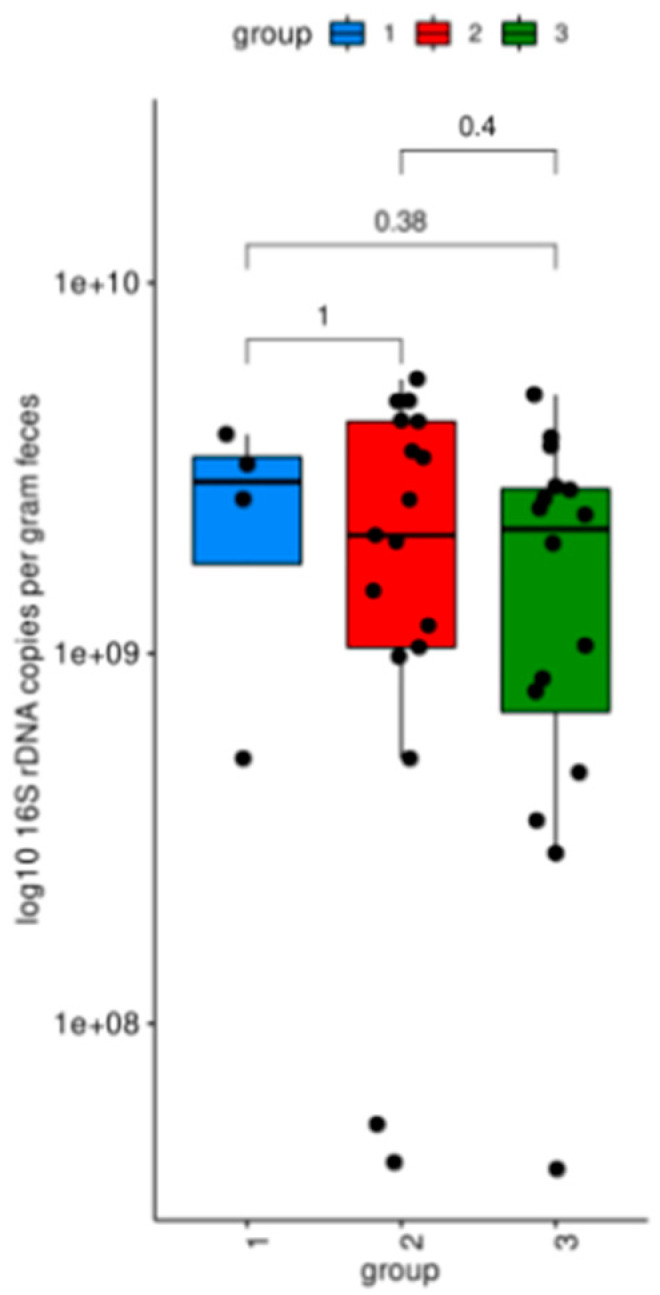
Total bacterial 16S rDNA copies. Group 1 (blue, no therapy); group 2 (red, teriflunomide); group 3 (green, other therapies).

For gut microbiome analysis, MS patients were grouped according to the DMT: treatment with teriflunomide (*n* = 17), other therapies (natalizumab, ocrelizumab, or fingolimod, *n* = 16), and no therapy (*n* = 4). The total bacterial load of the intestinal microbiota was assessed by real-time PCR quantification of bacterial 16S rDNA copies. No significant difference in bacterial load was observed between the three different treatment groups (Figure 1).

**Figure 2:**
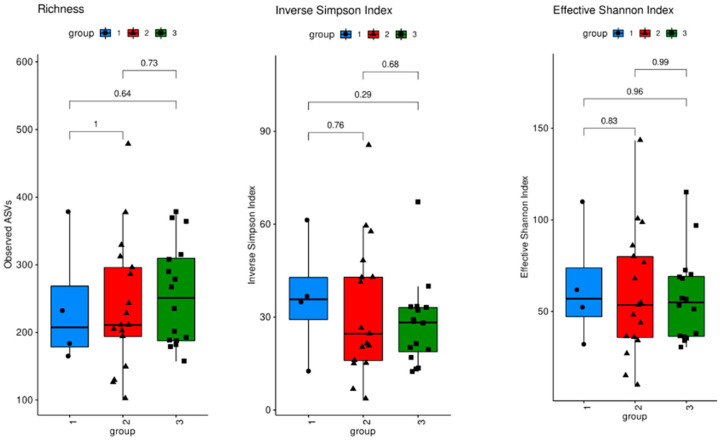
Alpha diversity: V1V3 regions of 16S rDNA. Group 1 (blue, no therapy); group 2 (red, teriflunomide); group 3 (green, other therapies).

Sequencing analysis of V1V3 regions of 16S rDNA was performed to assess the diversity and distribution of bacterial taxa. All three measures of alpha diversity assessed (Richness, the Inverse Simpson Index, and the Effective Shannon Index) showed no significant differences between the three groups of MS patients (Figure 2). No differences in bacterial compositions between the treatment groups were observed (Supplementary Figure S1). The level of relative abundance of *Clostridiaceae* (where Clostridium perfringens is classified) was very low in all groups. Additionally, no differences in relative abundance of bacteria were observed on the genus level (Supplementary Figure S2). 

**Figure 3:**
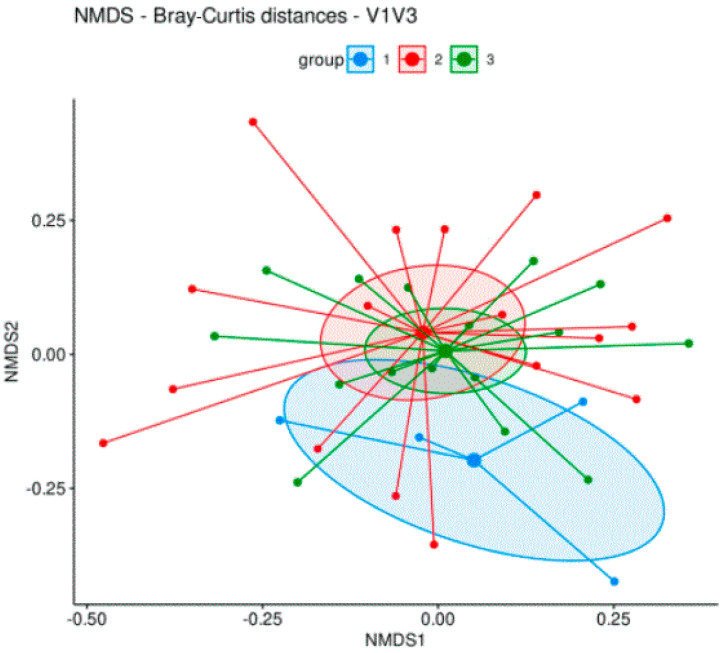
Beta diversity: Bray–Curtis distances. Group 1 (blue dots, no therapy); group 2 (red dots, teriflunomide); group 3 (green dots, other therapies). Large dots indicate the centroid of each group. Ellipses mark the 95 percent confidence intervals around each centroid.

**Figure 4:**
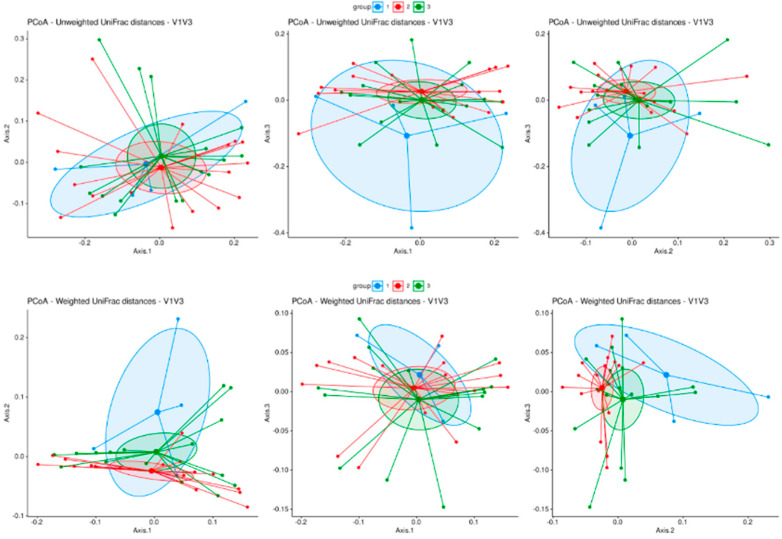
Beta diversity principal coordinate analysis of bacterial bd (including phylogenetic distances): unweighted UNIFrac distance and weighted UNIFrac distance. Group 1 (blue dots, no therapy); group 2 (red dots, teriflunomide); group 3 (green dots, other therapies). Large dots indicate the centroid of each group. Ellipses mark the 95 percent confidence intervals around each centroid.

Moreover, the evaluation of ordinated Bray–Curtis dissimilarities (Figure 3) and unweighted as well as weighted UNIFrac distances (Figure 4) followed by PERMANOVA analysis revealed no significant difference in beta diversity between the three groups. The evaluation of differential abundance between all groups using linear discriminant analysis (LefSE) revealed no significant features.

### 3.2. Epsilon Toxin in Serum

#### 3.2.1. Direct Detection of Epsilon Toxin

No sample showed a positive signal for epsilon toxin using the commercially available qualitative epsilon toxin assay, while positive control samples spiked with epsilon toxin at concentrations of 25 and 2.5 ng/mL gave strong signals. With the highly sensitive immunoassay [19], no quantifiable levels of epsilon toxin in MS patient serum could be detected. This direct sandwich ELISA reached a limit of detection of 1.1 ± 0.1 pg/mL and limit of quantification around 3.9 ± 0.4 pg/mL and was performed three times in duplicate. Even though some signals were slightly above the limit of detection, no serum with a quantitative positive signal was identified (Supplementary Table S1). As the accessibility of epsilon toxin to detection antibodies is a prerequisite to detection by sandwich ELISA, epsilon toxin complexed with endogenous antibodies in serum could possibly interfere with the direct detection of the toxin in serum by ELISA. Different combinations of physical, chemical, and mechanical approaches for the dissociation of Ig/epsilon toxin immunocomplexes were tested but no irreversible dissociation of immunocomplexes in human serum could be achieved.

#### 3.2.2. Detection of Immunoglobulin/Epsilon Toxin Immunocomplexes

A sandwich ELISA was performed to detect immunocomplexes. The assay was performed twice in duplicate, and one serum sample (1/38, 2.6%) yielded a slightly positive signal in both tests. However, high variability was observed among five commercial human control sera that were applied as negative controls and the addition of variable concentrations of epsilon toxin to patient sera did not result in competition in the ELISA, arguing against a specific detection of Ig/epsilon toxin immunocomplexes in human serum under the analyzed conditions.

#### 3.2.3. Detection of Anti-Epsilon-Toxin Antibodies

Furthermore, immunoassays were performed to detect anti-epsilon toxin antibodies. Four antibody combinations were used to analyze IgG, IgM, and IgA and each test was repeated twice in duplicate. Several signals above the limit of quantification were identified, of which most could not be reproduced in the second test series (Supplementary Table S1). Sera from six MS patients showed reproducible low immunoreactivity against epsilon toxin (4/38, 11%), which was regarded as a non-specific signal, or as part of a polyspecific immune response known to occur in chronic inflammatory diseases of the CNS.

## 4. Discussion

Microbial dysbiosis and its role in the development of diseases have gained growing interest and it has been suggested that the gut microbiota may play a role in the pathogenesis of MS [5,10,11]. This study aimed to gain further insight into the gut microbiome of MS patients, the presence of Clostridia in it, the occurrence of epsilon toxin in MS patients, and the impact of immune modulatory treatment on both gut microbiota and epsilon toxin levels.

Microbiome analysis, using 16S rRNA sequencing, revealed no significant differences in the quantity, diversity, or composition of the fecal microbiota between MS patients receiving teriflunomide treatment, other therapies, or no therapy. Parameters of alpha and beta diversity were investigated to identify differences in the microbial community structure within individual samples and between the different samples. 

The total bacterial load of the gut microbiota was similar between the three groups, indicating that there were no significant differences in the absolute bacterial abundance among the groups. Measures of alpha diversity showed no significant differences in the microbial community structure within individual samples. No significant differences were observed in the relative abundance of specific bacterial families or genera among the groups. Moreover, the evaluation of beta diversity revealed no significant difference between the three groups, suggesting a similar microbial community structure of MS patients who received teriflunomide treatment, other therapies, or no therapy. 

The relative abundance of *Clostridiaceae*, where *C. perfringens* is classified, was very low in all groups. It has been suggested that DMTs may reduce the abundance of *C. perfringens* [13]. Most analyzed patients received a DMT. We were especially interested in the impact of teriflunomide treatment on the microbial abundance of Clostridium perfringens and hence on epsilon toxin levels in serum. However, due to the limited number of patients in this pilot study, no statement on the influence of teriflunomide treatment or DMT in general on microbial composition and on the abundance of *C. perfringens* can be made. Taken together, the investigated groups of MS patients were not significantly different regarding their microbiome composition.

Recently, it was suggested that *C. perfringens* epsilon toxin might play a causative role in the etiopathogenesis of multiple sclerosis [10,11]. To confirm an exposure to epsilon toxin in the examined cohort of MS patients, different approaches were considered, including the direct detection of epsilon toxin in serum as well as the demonstration of immunoreactivity to epsilon toxin via the detection of anti-epsilon-toxin antibodies in blood or immunocomplexes made of epsilon toxin and serological antibodies. Previous studies suggested a contribution of *C. perfringens* to the cause of MS based on immunoreactivity to epsilon toxin [10,11]. However, the concomitant polyspecific immune response, which is a hallmark of MS, is not dependent on the presence of a causative antigen and is accompanied by immunoreactivity to a plethora of antigens [21,22]. Identified target antigens include neurotropic viruses and microorganisms as well as various self-antigens [21,22,23,24]. The MRZ(-H) reaction, i.e., an intrathecal polyspecific antibody synthesis directed against neurotropic measles, rubella, varicella zoster, and/or herpes simplex viruses is a very specific biomarker for MS and few other chronic inflammatory autoimmune diseases that affect the CNS [25]. 

As unspecific immunoreactivity against various antigens is known to occur in MS patients and does not necessarily indicate a primary immune response against a disease-related agent, the present study aimed to directly detect epsilon toxin in serum samples. The detection of epsilon toxin has been reported to be challenging, and to the best of our knowledge, there is only one commercially available assay for its detection in human material. Previous studies investigating immunoreactivity to *C. perfringens* epsilon toxin employed conventional Western blot techniques to detect anti-epsilon-toxin antibodies, with a methodological limitation of limited specificity [10,11]. Besides the application of the commercial assay, samples additionally were sent to Service de Pharmacologie et d’Immunoanalyse, where a highly sensitive immunoassay for the detection of epsilon toxin in human biological samples was established and extensively characterized [19]. Among the analyzed samples, there was no evidence for epsilon toxin in MS patient sera. However, it is possible that epsilon toxin is complexed with human serologic antibodies, which could potentially impact or prevent its accessibility to detection by sandwich ELISA. 

In addition to the approach of direct epsilon toxin detection, patient samples were tested for the presence of serological antibodies to the toxin. Four antibody pairs were used to detect IgG, IgM, and IgA specific to the toxin. Even though a total of 10/38 samples yielded a positive result in at least one assay, the overlap and especially reproducibility of the results was limited (Supplementary Table S1), and all positive signals were slightly above the determined limit of detection of the respective sandwich ELISAs. Based on the available results, legitimate doubts can be raised on the reproducibility and specificity of the detection of immunoreactivity against epsilon toxin in human serum and results should be interpreted with caution. 

As for the direct detection of epsilon toxin in serum, the detection of epsilon toxin-specific immunoglobulins also requires accessibility to detection by the used antibodies, which could be impaired or prevented by the formation of epsilon toxin/immunoglobulin complexes in serum, either in vivo or in vitro. Immunocomplexes were analyzed by sandwich ELISA. Even though one patient sample showed a slightly positive signal in both test replicates, the variations observed in the signals of the commercial human sera applied as negative controls required to assess the specificity of the measured signals in patient samples. For specificity assessment, variable concentrations of epsilon toxin were added to patient sera. As no competition was observed by the hook effect, the results were not in favor of a specific detection of immunocomplexes involving epsilon toxin.

Taken together, the results of this study did not provide evidence supporting the hypothesis of a causal relationship between *C. perfringens* epsilon toxin and MS. In the past, many viral and non-viral agents have been discussed or postulated as possible causes of MS. So far, no infectious agent could be linked to MS pathogenesis in a direct causal relationship. Also, in the currently promising Epstein–Barr virus (EBV) hypothesis, indirect immunological mechanisms are assumed to drive the development of MS [26,27].

The limitations of this study are the small sample size and the small size of its subgroups, especially the number of four untreated MS patients, which does not allow for a comparison between treatment-naïve MS patients and patients receiving DMT. Furthermore, microbiota analysis was restricted to stool samples rather than gastrointestinal samples. Even though it is a common, non-invasive, and user-friendly sampling method, there are several limitations to this approach, as fecal samples are not representative of the comprehensive gut microbiota and bias might be introduced by environmental and pre-analytical factors [28]. The inclusion of both immunoreactivity analysis and direct analysis of epsilon toxin as well as toxin/Ig immunocomplexes is a strength of the study. Additionally, the use of both a commercial and in-house assay for the detection of epsilon toxin in blood enhances the robustness of the investigation.

## 5. Conclusions

In this pilot study analyzing the gut microbiota and sera of patients with MS, we could not provide evidence supporting the hypothesis of a causal relationship between *Clostridium perfringens* epsilon toxin and MS.

Furthermore, a workflow for gut microbiome analysis and stool self-sampling of patients was developed, paving the way for future studies including more patients. With regard to the analysis of the gut microbiome and serum for the detection of epsilon toxin and anti-epsilon toxin, this study was limited due to its sample size and did not yield significant findings. 

## Data Availability

Raw data can be provided upon reasonable request by the corresponding author.

## Supplementary Materials

The following supporting information can be downloaded at: https://www.mdpi.com/article/10.3390/biomedicines12071392/s1, Figure S1: Family abundance; Figure S2: Genus abundance; Table S1: Serological analyses for the detection of epsilon toxin and epsilon toxin immunoreactivity.
