# Supplementary material for: Multiple Sclerosis and Clostridium perfringens Epsilon Toxin: Is There a Relationship?
Source: Biomedicines. 2024 Jun 23;12(7):1392. doi: 10.3390/biomedicines12071392 (PMC11274216; doi:10.3390/biomedicines12071392)
Supplement: Supplementary file 1 [file biomedicines-12-01392-s001.zip › biomedicines-3023293-supplementary.pdf]

Multiple Sclerosis and *Clostridium perfringens* Epsilon Toxin: Is There a Relationship?

André Huss [1,†], Franziska Bachhuber [1,†], Cécile Feraudet-Tarisse [2], Andreas Hiergeist [3] and Hayrettin Tumani [1,*]

[1] Department of Neurology, University Hospital Ulm, 89081 Ulm, Germany
[2] CEA, INRAE, Medicines and Healthcare Technologies Department (DMTS), SPI, Paris-Saclay University, 91191 Gif-sur-Yvette, France
[3] Institute of Clinical Microbiology and Hygiene, University Medical Center, 93053 Regensburg, Germany
* Correspondence: hayrettin.tumani@uni-ulm.de
† These authors contributed equally to this work.

Supplement

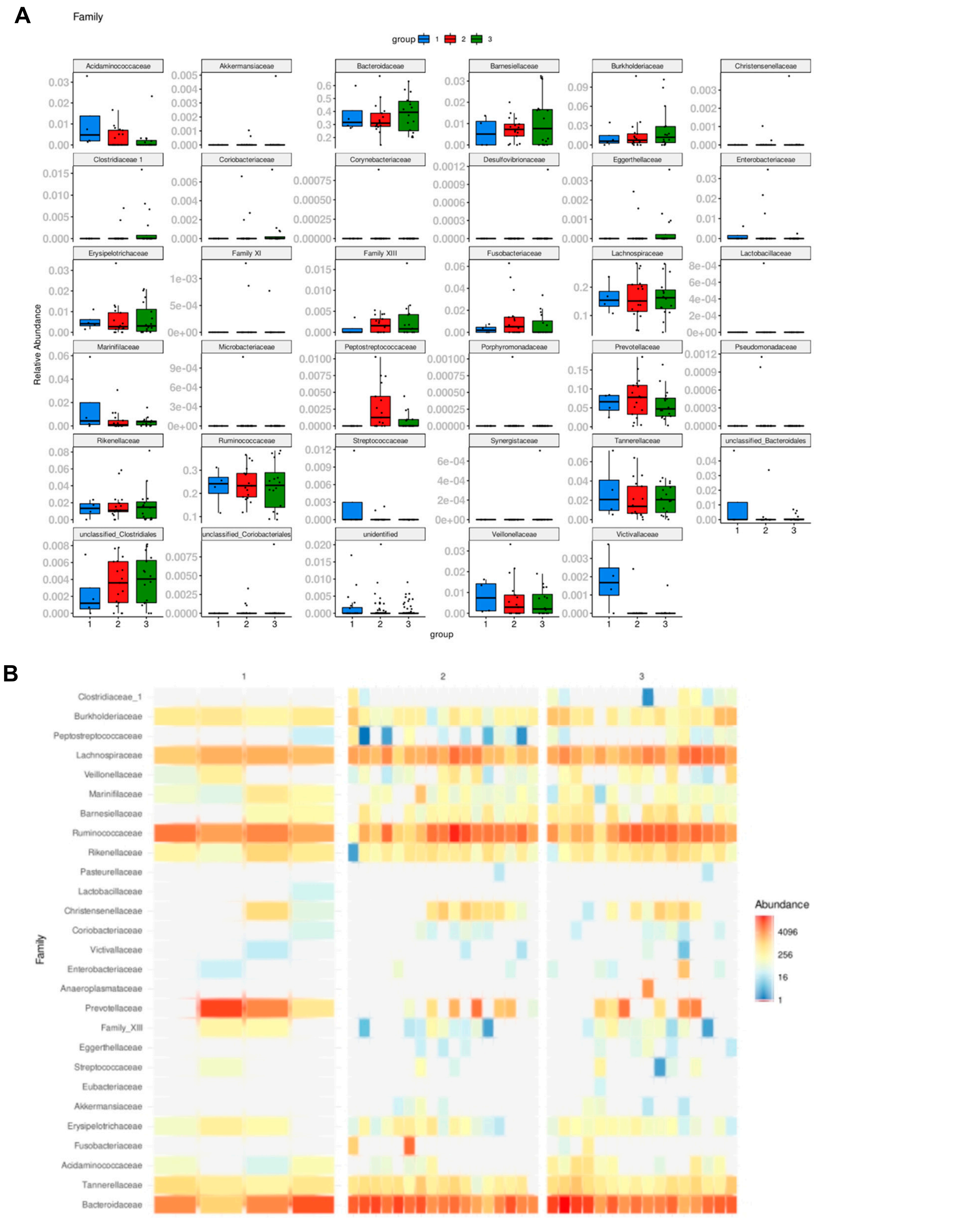

Supplementary Figure S1: Family abundance. Relative abundance on the family level (A). Family abundance heatmap (B).

A

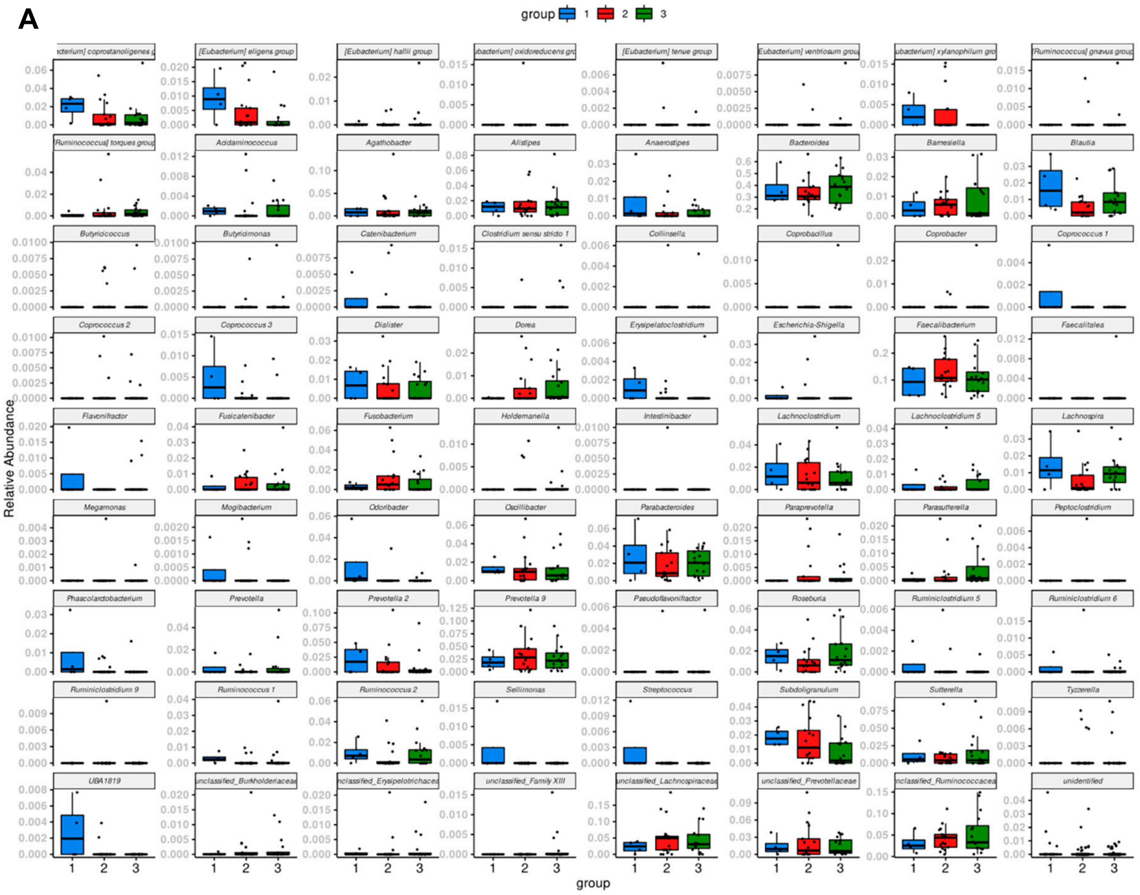

group 1 2 3
Relative Abundance
group
[Eubacterium] eligens group
[Eubacterium] hallii group
[Eubacterium] tenue group
[Ruminococcus] gnavus group
[Ruminococcus] torques group
Acidaminococcus
Agathobacter
Alistipes
Anaerostipes
Bacteroides
Barnesiella
Blautia
Butyricicoccus
Butyricimonas
Catenibacterium
Clostridium sensu stricto 1
Collinsella
Coprobacillus
Coprobacter
Coprococcus 1
Coprococcus 2
Coprococcus 3
Dialister
Dorea
Erysipelatoclostridium
Escherichia-Shigella
Faecalibacterium
Faecalitalea
Flavonifractor
Fusicatenibacter
Fusobacterium
Holdemanella
Intestinibacter
Lachnoclostridium
Lachnoclostridium 5
Lachnospira
Megamonas
Mogibacterium
Odoribacter
Oscillibacter
Parabacteroides
Paraprevotella
Parasutterella
Peptoclostridium
Phascolarctobacterium
Prevotella
Prevotella 2
Prevotella 9
Pseudoflavonifractor
Roseburia
Ruminiclostridium 5
Ruminiclostridium 6
Ruminiclostridium 9
Ruminococcus 1
Ruminococcus 2
Sellimonas
Streptococcus
Subdoligranulum
Sutterella
Tyzzerella
UBA1819
unclassified_Burkholderiaceae
unclassified_Family XIII
unclassified_Lachnospiraceae
unclassified_Prevotellaceae
unidentified

B

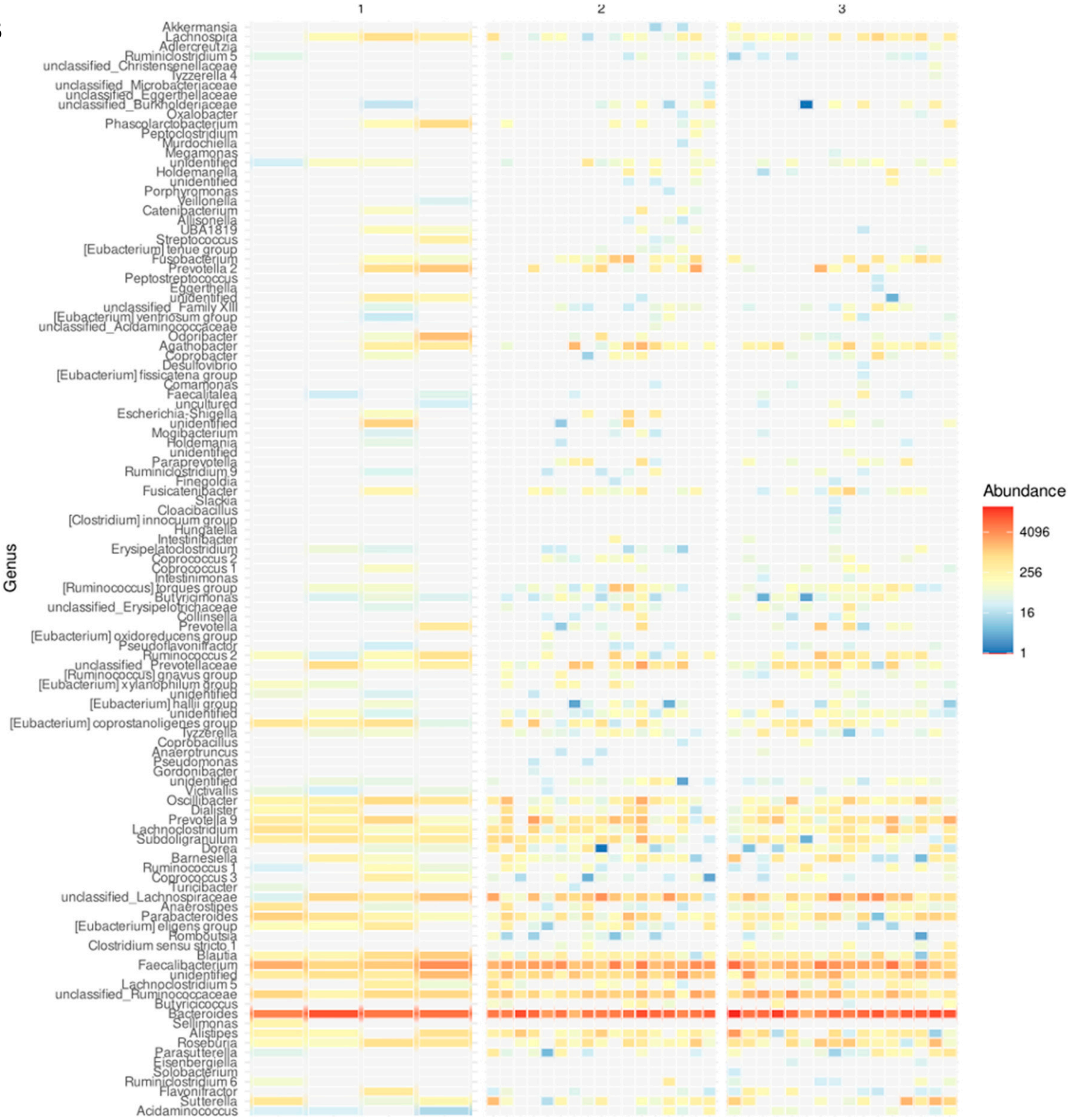

Supplementary Figure S2: Genus abundance. Relative abundance on the genus level (A). Genus abundance heatmap (B).

Supplementary Table S1: Serological analyses for the detection of epsilon toxin and epsilon toxin immunoreactivity. 38 Serum samples from 34 patients were analyzed. Direct detection of epsilon toxin was performed by a commercial qualitative immunoassay (Elisa A) as well as an in-house assay (Elisa B). Number (#) of assay replicates is indicated. For detection IgG specific for epsilon toxin, two different antibody pairs were used. IgG (a): IgG1, IgG3, IgG4. IgG (b): IgG1, IgG2, IgG3, IgG4.

**Patient**	**Epsilon toxin**				**Anti-epsilon toxin AB**								**Ig/epsilon toxin immuno-complexes**	
	Elisa A	Elisa B			IgG (a)		IgG (b)		IgM		IgA			
		# 1	# 2	# 3	# 1	# 2	# 1	# 2	# 1	# 2	# 1	# 2	# 1	# 2
**01 (V1)**							+		+	+				
**01 (V2)**							*		+	+				
**01 (V3)**							*		+	+				
**02 (V1)**									*					
**02 (V2)**														
**03**			*						*	*				
**04**														
**05**														
**07 (V1)**						*		*						
**07 (V2)**						*								
**08**									+	*				
**09**									*					
**11**									*	*				
**14**														
**16**									+	*				
**18**										*				
**19**							*			*			*	
**20**									*					
**21**						*		*	*					
**23**						+		+						
**24**									+	+		*		
**25**														
**26**						*		+	*				*	*
**27**		*												
**28**												*		*
**29**									*					
**30**									*	*				
**31**									*	*	+			
**32**						*			+					
**33**					*									
**34**														
**35**														
**36**														
**37**							*							
**38**														
**39**										*				
**40**														*
**41**												*		

positive

Number (of patients)	0/34	0/34	0/34	0/34	0/34	1/34	1/34	2/34	5/34	2/34	1/34	0/34	0/35	0/34
% (of patients)	0	0	0	0	0	3	3	6	15	6	3	0	0	0

+ Positive signal above limit of quantification. * Signal above limit of detection, below limit of quantification. V: visit.
